# Structural Analysis and Epitope Prediction of MHC Class-1-Chain Related Protein-A for Cancer Vaccine Development

**DOI:** 10.3390/vaccines6010001

**Published:** 2017-12-25

**Authors:** Tayo Alex Adekiya, Raphael Taiwo Aruleba, Sbonelo Khanyile, Priscilla Masamba, Babatunji Emmanuel Oyinloye, Abidemi Paul Kappo

**Affiliations:** 1 Biotechnology and Structural Biology (BSB) Group, Department of Biochemistry and Microbiology, University of Zululand, KwaDlangezwa 3886, South Africa; adekiyatalex@gmail.com (T.A.A.); arulebataiwo@yahoo.com (R.T.A.); s7khanyile@gmail.com (S.K.); presh4u@rocketmail.com (P.M.); tunji4reele@yahoo.com (B.E.O.); 2 Department of Biochemistry, College of Sciences, Afe Babalola University, PMB 5454, Ado-Ekiti 360001, Nigeria

**Keywords:** antigenic peptides, bioinformatics, cancer, MIC-A, vaccine, 3-D structure, epitopes

## Abstract

Major histocompatibility complex class 1 chain-related gene sequence A is a polymorphic gene found at about 46.6 kb centromeric to HLA-B. It encodes a transmembrane protein, which is a non-classical human leukocyte antigen whose expression is normally induced by stress conditions like cancer and viral infections. The expression of MIC-A leads to the activation of NKG2D receptors of natural killer and T cells, leading to the generation of innate immune response that can easily eliminate/cleanse tumour cells and other cells that express the protein. Several bioinformatics and immunoinformatics tools were used to analyse the sequence and structure of the MIC-A protein. These tools were used in building and evaluating modelled structure of MIC-A, and to predict several antigenic determinant sites on the protein. The MIC-A protein structure generated an average antigenic propensity of 1.0289. Additionally, the hydrophilic regions on the surface of the MIC-A protein where antibodies can be attached were revealed. A total of fourteen antigenic epitopes were predicted, with six found in the transmembrane protein topology, and are predicted to play a role in the development of vaccines that can reactivate the functionalities of the MIC-A protein on the surface of cancer cells in order to elicit a desired immune response.

## 1. Introduction

Cancer is one of the most common and widely diagnosed diseases around the world. It is caused by unusual cell growth or cell division and has the ability to invade or spread to other parts of the body from the point of tumour formation (otherwise known as primary tumours). This has been reported to be the leading cause of death in our modern society [1]. Currently, different therapies on how to combat this deadly disease has been built on developing prognostic and predictive tools based on the status of lymph nodes, the size and grade of tumour, hormone receptors, and human epidermal growth factor receptor 2 (HER2) expression [2]. However, these therapies have been considered ineffective in treating cancer. It has been observed and demonstrated that the immune system response plays a key role in the generation, progression, proliferation, and spread of tumours [3]. Certain actions of the immune system may lead to the detection of tumour cells during tumour formation, which can easily lead to the abolishment and cleansing of tumours [3,4]; though tumour cells may display various characteristics which make it difficult for the immune system to recognize them due to their unstable genetic nature [5].

Hence, the activation of the NKG2D receptor of natural killer cells when connected to its ligands, which is induced as a result of infections and other inducers of cellular stresses [6,7], such as major histocompatibility complex class I chain-related proteins sequence A and B (MIC-AB) and unique long 16 (UL16) binding proteins 1–6 (ULBP1–6), show great immunotherapy and immunosurvelliance effects on tumours [8,9,10]. The activation of these NKG2D receptors has been observed to have a stimulatory effect on natural killer cells, NKT cells, gδ^+^ T cells, and CD8^+^ T cells [11]. Hence, it is believed that NKG2D ligands trigger the action of immune system responses to combat and kill tumours, which expresses such ligands, hence, serving as anti-tumour agents [10,11].

Failure in regulating NKG2D ligands, either by downregulation or shedding of ligands from the surface of the cell and loss in the ability of proteins that help in activating immune responses (e.g., human leukocyte antigen class I; HLA) or upregulation of proteins responsible for the loss in the activation of immune response (such as non-classical human leukocyte antigen-I), have been found in various kinds of cancers [12,13]. Therefore, this present study focused on the sequence and structural analysis, as well as the immunoinformatic investigation of the hydrophobicity, antigenicity, surface accessibility, and epitopes location of major histocompatibility complex class I chain-related protein A (MIC-A), which is one of the NKG2D ligands that activate the NKG2D receptors on natural killer cells as well as T-cells that serve as innate immunity against tumour growth. Additionally, this study was carried out to form the basis for subsequent studies that will lead to the development of a therapeutic vaccine against cancer.

## 2. Materials and Methods

### 2.1. Sequence Retrieval and Comparative Modelling

The NCBI database [14] was searched to retrieve the coding sequence of the major histocompatibility complex class 1 chain related protein A (MIC-A). The sequence ID of MIC-A is KY500939.1 and the mRNA sequence consisting of the coding region (exons), excluding the non-coding regions (introns), was obtained. Subsequently, the mRNA sequence was translated into an amino acid sequence via the translate tool on the ExPASy server [15]. Thereafter, several bioinformatics and computational tools, as well as databases, were used to query various properties, such as physicochemical, functional, structural characterization, and antigenic determinants or epitopes in the MIC-A protein were ascertained.

### 2.2. Primary and Secondary Structural Prediction

In the prediction of MIC-A primary structure, the following physicochemical parameters was predicted by protparam tool from ExPASy (web.expasy.org/protparam/): number of amino acids, molecular weight, amino acid composition, atomic composition, theoretical PI, extinction coefficient, estimated half-life, instability index, aliphatic index, and grand average of hydropathicity (GRAVY) [16]. The secondary structure of MIC-A was predicted using PSI-blast-based secondary structure PREDiction (PSIPRED, bioinf.cs.ucl.ac.uk/psipred/) which is a simple and precise method to predict the secondary structure of a protein [17,18].

### 2.3. Homology Modelling of MIC-A Protein

Homology modelling predicting the three-dimensional (3D) structure of the MIC-A protein was conducted using the Swiss-model tool found on the ExPASy website https://swissmodel.expasy.org/ [19]. This is an automated server which integrates a web-based modelling system to generate a homology model for a protein. A library of experimentally-determined protein structures was searched to identify suitable templates. The structure template with the PDB ID 1hyr.1.C MHC class 1 chain-related protein A was selected for the query protein sequence and this was used as a reference in determining the 3D structure of the query protein [20]. Thereafter, the 3D model result generated in a PDB format was analysed and visualized using PyMOL version 1.3 available at https://pymol.org/2/ [21].

### 2.4. Model Evaluation and Stereochemical Analysis

Following the generation of the 3-D model, the stereochemical analysis and structural evaluation of the model were accomplished using several validation and evaluation tools. The assessment of the backbone conformation was done using psi/phi Ramachandran plot, which determines whether stereochemical parameters have been violated during model building [22]. The main reason for measuring psi against phi torsion angles is because these angles could rotate and, in turn, lead to unique structures, like the α-helix and β-sheets. The accumulation of bulky side chains of some hydrophobic amino acids may result in steric hindrance, leading to poor quality of a modelled structure. Thus, the Ramachandran plot of the phi/psi distribution in the 3D model of MIC-A protein is developed by RAMPAGE (mordred.bioc.cam.ac.uk/~rapper/rampage.php). Moreover, the overall quality of the model was determined by QMEAN [23], which shows that the 3-D structure of the protein ranges within the scores typically found in native proteins of matching size.

### 2.5. Antigenic Epitope Prediction

The position of antigenic epitopes present on the MIC-A protein sequence was determined using the antigenic peptide prediction tool on the bioinformatics server of Immunomedicine Group of the Universidad Complutense de Madrid [24]. This server uses the method described by Kolaskar and Tingankar [25] to predict the segments within a protein sequence which are likely to be antigenic; capable of eliciting an antibody response. The accuracy of this method is about 75% in predicting the antigenic epitopes of protein sequence. The algorithm uses four steps in predicting the antigenic determinants of a protein; in the first step, the average antigenic propensity value <Ap> was calculated for each of the overlying heptapeptides, starting from the N-terminal to C-terminal of the protein. These generated average values were allocated to the fourth residue (i + 3) in the segment, where the average antigenic propensity value of the protein was determined; this was step two. In the third step, the residues having <Ap> greater than or equal to 1.0 were termed as possible antigenic residues. Therefore, to pick antigenic determinants for step four, a condition was set where six repeated residues had to satisfy the third step [25].

After the subsequent prediction of possible antigenic epitopes present in MIC-A, the transmembrane topology of all the predicted epitopes were determined using TMHMM version 2.0 found at http://www.cbs.dtu.dk/services/TMHMM/ [26], which shows whether the epitopes were present in transmembrane regions or not. TMHMM, works based on the hidden Markov model (HMM) technique, which specializes in modelling various regions of a membrane proteins, such as helix caps, globular domains, the middle of a helix, as well as regions close to the membrane [26].

Furthermore, the hydrophobicity of the protein sequence was done using ExPASy ProtScale tool https://web.expasy.org/protscale/ [16], which analysed the physicochemical behaviour of the antigenic sequences, as well as identified several highly hydrophilicity regions. The program works by using a moving-segment method that continuously depicts the average hydropathy within a segment of predetermined length as it moves through the sequence. The successive scores are plotted from the N-terminus to the C-terminus. At the same time, a line at the centre is printed which corresponds to the grand average of hydropathy of the amino acid compositions that are found in the most sequenced proteins [16].

## 3. Results

### 3.1. Primary and Secondary Structure of the MIC-A Protein

The sequence of the MIC-A protein was retrieved from the NCBI protein database using accession number KY500939.1. This sequence of interest was generated after various sequences that have significant alignments with the sequence in question were retrieved. Approximately, twenty-one (21) sequences in conjunction with the query sequence were selected based on their E-values. This refers to the number of times the database match might have occurred randomly, thus providing identity values that ranges from 80% to 100%. Hence, the sequence with the lowest E-value was chosen due to the fact that it is actually the sequence with the highest validity. From the primary structure of the protein, the MIC-A protein had an estimated molecular weight of 42,915.40 Da (~43.0 kDa), theoretical isoelectric point of 6.49. This indicates that the protein is negatively charged due to its isoelectric point, which is below 7, as well as the presence of a high proportion of negatively-charged amino acid residues against the number of positively-charged residues, which are 41 and 37, respectively. The instability index of the protein is shown to be 49.59, which is indicative of the protein being unstable in solution. The negative grand average of hydropathicity (GRAVY) of −0.429 indicates that the protein was hydrophilic and, hence, can interact with the aqueous environment or the amide water [27].

**Figure 1:**
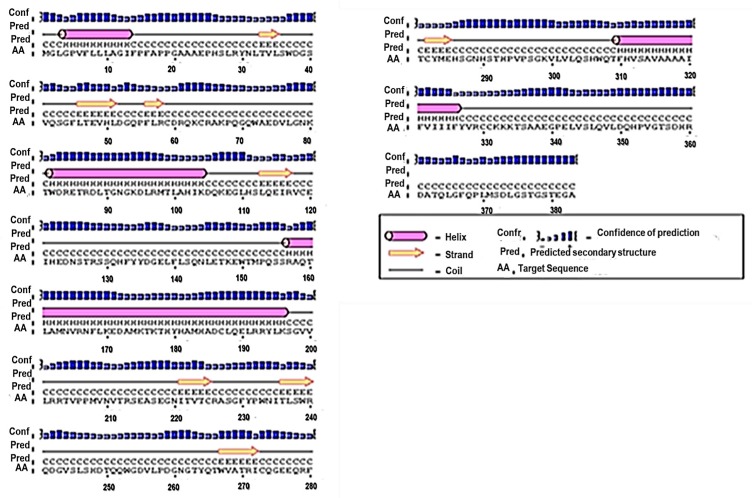
Predicted secondary structure of MIC-A. Using PSIPRED, the secondary elements in the protein were predicted to consist of four α-helices and eight β-sheets.

PSIPRED is a secondary structure prediction method that integrates two neutral feed-forward networks, which perform output analysis acquired from PSI-BLAST [17,18]. The predicted structure of the protein was achieved by submitting the FASTA sequence of the MIC-A protein to obtain a graphical representation of the secondary structure of the protein through http://bioinf.cs.ucl.ac.uk/psipred/. The secondary structure predicted by PSIPRED revealed that the protein had four α-helices which are represented by the pink cylindrical structures, while the eight yellow arrows represent extended β-strands, as shown in Figure 1.

### 3.2. Homology Modelling of MIC-A Protein

**Figure 2:**
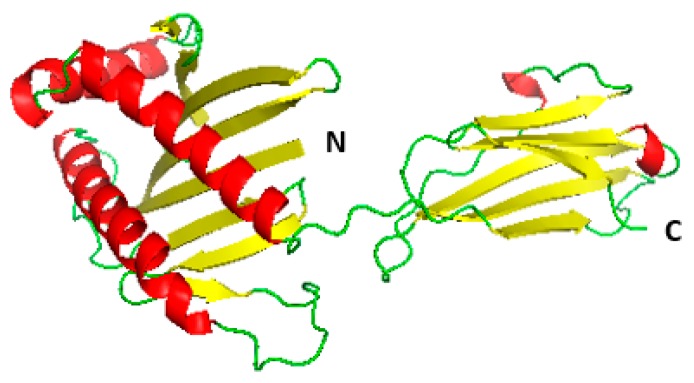
Predicted 3D model structure of MIC-A. The structure depicts three standard helices and two 310 helices, as well as 14 antiparallel β-sheets containing a short β-strand. The positions of the N and C-terminal of the protein are also shown.

In homology modelling, the first and basic step is to identify the best corresponding template using similarity-searching programs, such as PBI BLAST, against the PDB database. The template is selected based on the sequence similarity to the query protein sequence. The protein with the PDB ID: 1hyr.1.C was selected as the template for the homology modelling of the MIC-A protein, out of the total 50 templates that were generated during the alignment or related-sequence search. Both the template and target protein sequence were used to predict the 3D model (Figure 2) of the query protein based on their sequence identities using the Swiss model workspace [20].

### 3.3. Model Evaluation and Stereochemical Analysis

**Figure 3:**
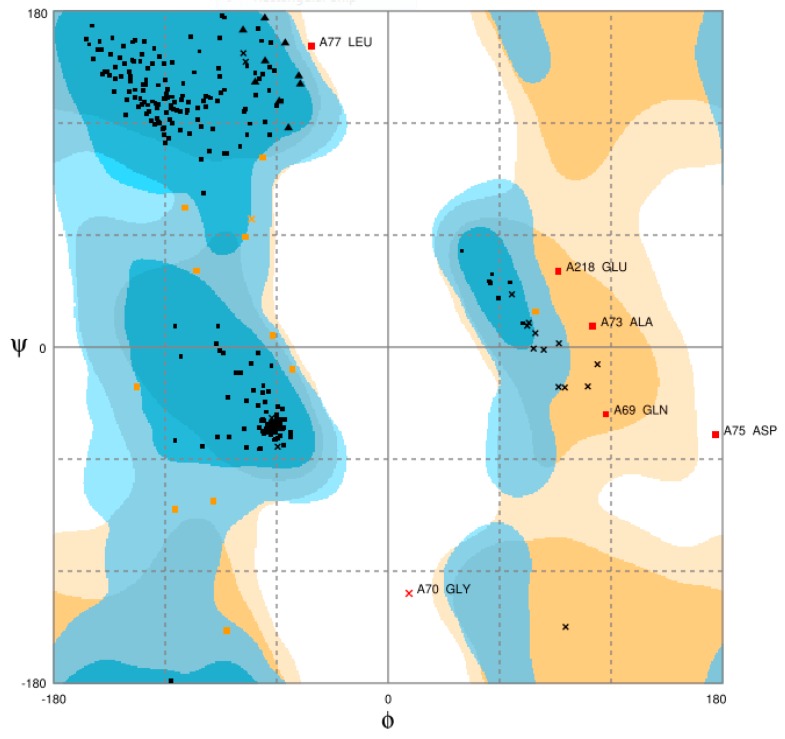
Ramachandran plot of the modelled MIC-A. The percentage of amino acid residues in the most favoured, allowed, and outlier regions are indicative of the quality of the modelled MIC-A protein structure.

The modelled structure was evaluated by RAMPAGE and was used to generate a Ramachandran plot [28]. This was done in order to determine the reliability and the quality of the modelled structure, as well as to ascertain whether any stereochemical parameters were violated during modelling by analysing residue-by-residue geometry and complete structure geometry. This tool was used in determining the energetically stable conformations of the psi (ψ) and phi (Φ) torsion or dihedral bond angles for each amino acid in the structure. The results of the Ramachandran plot generated in Figure 3 showed 93.3% of residues were found in the favourable region of the plot and the number of residues in the allowed region was 4.4%. Conversely, 2.2% of the residues were found in the outlier region, and some of the residues include Leu77, Glu218, Ala73, Gln69, Asp75, and Gly70, which could hinder the quality of the modelled structure. The total percentage of the residues found in the favoured and allowed regions was 97.7%, making the model a convincing and believable model. The ideal result should be that 90% of the residues are present in the favoured region [29], however, the total number that was found in that region in this study exceeded the expected value.

**Figure 4:**
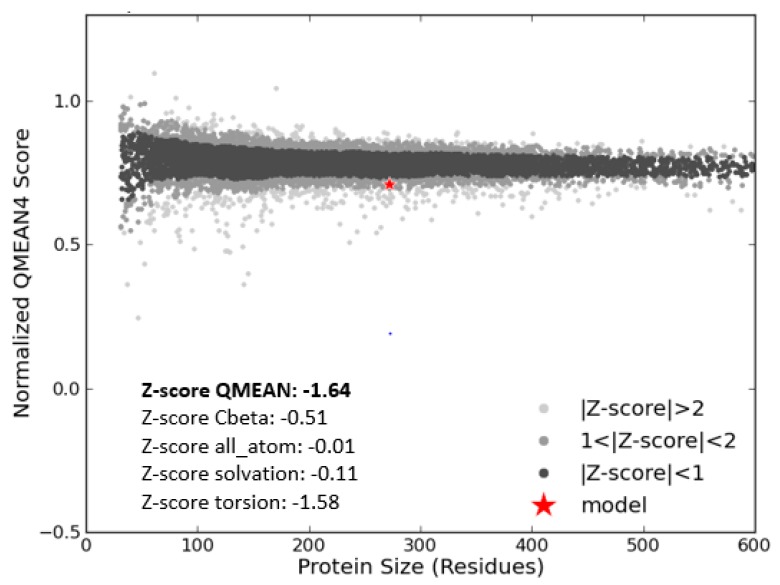
Structural quality of the modelled MIC-A protein. The Z-score of MIC-A protein is compared with non-redundant sets of PDB structures showing the quality of the modelled structure by the Swiss model.

The QMEAN score (Z-score) estimates the reliability of the predicted modelled 3-D structure [23]. The score of the protein was −1.64 as shown in Figure 4. Thus, the Ramachandran plot and QMEAN score results confirmed the quality of the 3-D model of MIC-A.

### 3.4. Prediction of Antigenic Epitopes on MIC-A Protein

**Figure 5:**
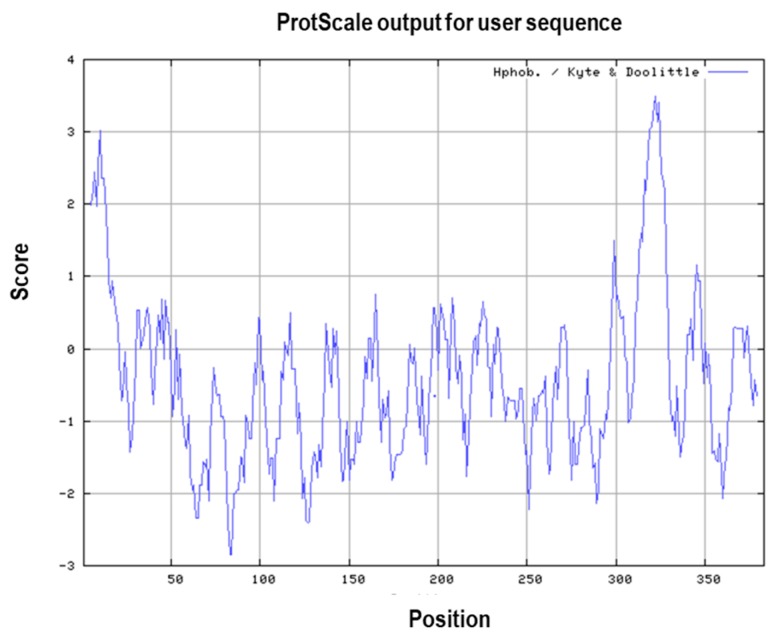
Kyte-Doolittle hydropathy plot for MIC-A. The window position values indicated on the *x*-axis of the graph reveals the average hydropathy of the entire window, with the corresponding amino acids as the middle element of that window. Plots above 0 (zero) in the graph indicate hydrophobic regions in the protein and those below 0 (zero) indicate the hydrophilic regions. Additionally, peaks with scores higher than 1.8, which is the threshold value for hydrophobic region, indicate possible transmembrane regions.

The hydrophobic property of MIC-A sequence was characterized and analysed using Kyte and Doolittle method [30] on ExPASy Protscale. This program uses a continuous moving-segment technique to determine the average hydropathy that is present in a segment of predetermined length as it moves through the sequence [30]. This method can be used in predicting regions of a protein that fall within or outside of a membrane, because the most optimal antigenic epitopes are hydrophilic, flexible, and are positioned on the surface of the protein. Furthermore, antibodies can only bind easily to epitopes that are found on the surface of a protein most especially when the epitopes are flexible, the antibodies bind with higher affinity and readily move into the most accessible positions [31,32]. As shown in Figure 5, the regions below the midpoint (zero) are within the interior of the protein, that is, they are hydrophobic in nature, while the regions above the midpoint are hydrophilic, which are likely to be exposed on the surface (outside) of a folded protein.

**Figure 6:**
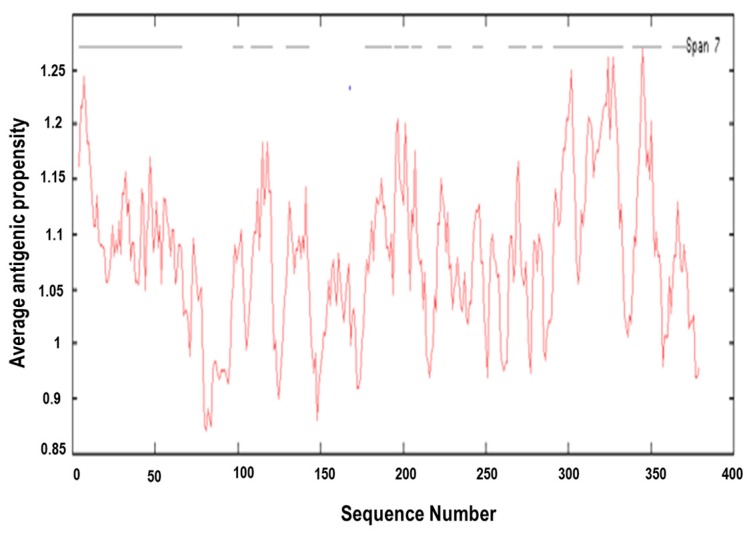
Antigenic plot of MMIC-A. The predicted peptides with potential antigenic properties depicting the start and end positions within the MIC-A sequence are shown.

**Figure 7:**
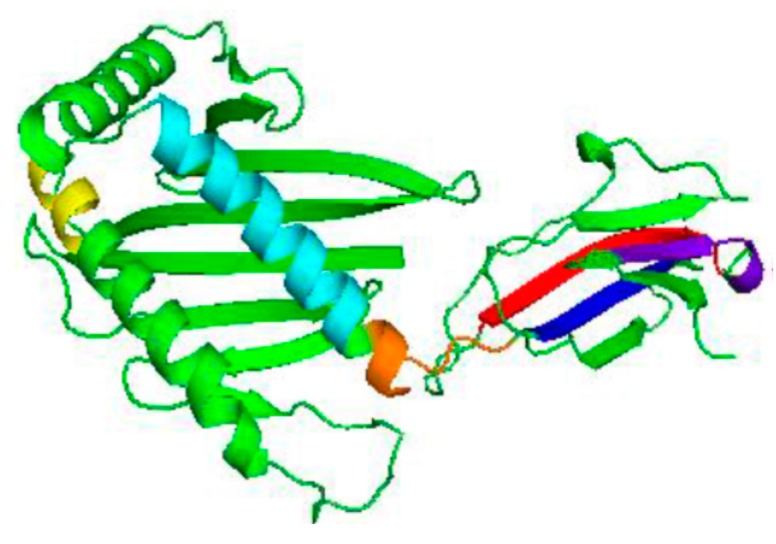
Structure of MIC-A showing the predicted epitopes in different colours. Depicted on the MIC-A protein structure are the predicted potential antigenic peptides in various colours, which are found in the transmembrane topology: the first, second, and third are represented by yellow, cyan and orange, while the fourth, firth, and sixth are shown in blue, red, and purple.

Figure 6 depicts an antigenic plot for MIC-A sequence. Additionally, the overall antigenic propensity score of MIC-A protein was predicted to be 1.0289, which is higher than the average score of 1.0 for potentially antigenic protein [24,25]. This indicates that the protein in question has a corresponding promising antigen. In total, 14 antigenic epitopes were predicted in the MIC-A sequence, which are specifically characterized by the peptides’ start and end positions as shown in Table 1. All predicted epitopes are displayed in Figure 7 with assorted colours. Thereafter, the transmembrane protein topology was determined using TMHMM, an online tool that predicted the transmembrane helices and location of the intervening loop regions of the protein [33].

The TMHMM results revealed that seven (7) of the predicted epitopes were exposed outside the transmembrane helices with lesser scores, which is an indication that the seven epitopes are non-antigenic. Meanwhile, one of the epitopes was found to be on the transmembrane helix, due to a high number of amino acids in the transmembrane helix, which has exceeded the expected value of eighteen (18). Additionally, the predicted transmembrane helix of this epitope could be a signal peptide due to high number of amino acids in the N-terminal of transmembrane helix [33]. Moreover, antigenicity was predicted to be found in six (6) epitopes, which falls within the transmembrane helices with high TMHMM scores, as shown in Table 1.

## 4. Discussion

In this study, we have examined the MIC-A protein whose expression in normal cells is unusual, but highly upregulated in stressed cells, virus-infected cells, and cancer cells [34]. Its expression in cancer cells has been shown to be responsible for the innate immune activities triggered by NK cells and T cells against abnormal growth. This protein has been shown to be a good prognosis tool due to its immunosurveillance activities in several types of cancers, such as breast, ovarian, and colorectal cancer [35]. However, failure in regulating this protein either by the downregulation or shedding of the protein on the surface of the tumour cells results in the loss of its activity in activating immune response.

In this study, we have analysed the protein sequence and studied the structure, as well as predicted the antigenic epitopes present in the MIC-A protein that are important in raising the desired immune response, which was accomplished using several types of highly precise bioinformatics tools. The primary structure of the MIC-A protein showed that leucine is the most abundant amino acid, making up approximately 9.40% of the total amino acids in the primary sequence followed by glycine, serine and threonine, which have equal amounts of amino acid residues (7.60%). The abundance of leucine and other non-polar amino acids in the MIC-A protein sequence contributes greatly to the stability of the 3-D shape and the hydrophobic nature of the protein. Hydrophobicity of a protein is one of the factors which determine its proper folding.

The 3D model of the query protein was built using a template sequence with ID number 1hyr.1.C found in the Swiss-model repositorym which has 100% similarity with the MIC-A sequence. Thereafter, QMEAN and RAMPAGE were used to ascertain the reliability and the overall quality of the modelled protein 3-D structure. The stereochemical parameters of the modelled structure were also ascertained in order to determine whether there is any structural violation during modelling. PyMol 1.3 version was used to visualise and evaluate, as well as analyse, the generated 3-D model [21]. Based on the fact that there is no existing vaccine against cancer, this present study was designed to give an insight towards therapeutic vaccine development against cancer through the prediction of protein-conserved residues, antigenic epitopes, and the secondary structure. Protscale predicted several antigenic epitopes which are hydrophilic, flexible in nature and are positioned on the surface of the protein which can move into the accessible positions where antibodies can easily bind to them with higher affinity [31,32]. In this study, the bioinformatics server of the Immunomedicine Group of the Universidad Complutense de Madrid was used to predict several antigenic epitopes present in the MIC-A protein. It can be deduced that these antigenic determinants are very important in raising the desired immune response [36]. Consequently, fourteen (14) epitopes were predicted in this study and seven of the total epitopes were found outside the transmembrane protein topology, while one of the epitopes was found to be a transmembrane peptide and the other remaining six epitopes were located inside the transmembrane protein. Epitopes in the transmembrane region cannot act as an antigen unless it contains a surface antigen which can create an intracellular N-terminus and does not contain any other known functional domain, such as a signal peptide [37]. Furthermore, it was discovered that out of the six epitopes that are found within the transmembrane protein topology, epitope YQTWVATRICQ at position 264 to 275 has the highest score of the N-terminal on the cytoplasmic side of the membrane, followed by QRFTCYM at position 278 to 284, and KTHYHAMHADCLQELRR at position 177 to 193; these epitopes returned higher scores than others and, hence, showed maximum antigenic peptide presence in the MIC-A protein. Therefore, these epitopes can be used in the design and development of a therapeutic vaccine that can activate immune response against cancer cells. This present study correlates with several other existing studies, such as Ashfaq and Ahmed [38], Rahman and Parvege [39], and Patwary and co-workers [40], who used several bioinformatics and immunoinformatics tools to depict several antigenic peptides which have the potential to elicit desired immune responses and can, hence, be used for vaccine design and development. However, in vivo and in vitro studies are imperative to confirm the generated results.

## 5. Conclusions

Bioinformatics and immunoinformatics are current and emerging interdisciplinary fields which use various techniques to counter the problem of time efficiency and cost expenditure when formulating new drugs or vaccines. Currently, no in vivo and in vitro studies have been carried out on this protein. In order to develop a therapeutic vaccine for cancer, it is of great significance to examine several antigenic components that are present in proteins which are responsible for the activation of cytotoxic activities through the stimulation of its NKG2D receptor. MIC-A has shown great potential in immunotherapy and immunosurvelliance against several kinds of tumours. Additionally, the shedding and the downregulation of MIC-A protein on the surface of tumour cells can be mediated by different biomolecules. Hence, this study has revealed six potential antigenic epitopes with higher scores at the N-terminal of the cytoplasmic region of the membrane. These epitopes can help in therapeutic vaccine development which can activate the activities of the MIC-A protein on the surface of cancer cells to raise desired immune response in cancerous patients.

## Figures and Tables

**Table 1:** Predicted antigenic epitopes within the MIC-A protein. A total of 14 epitopes are predicted, with six of those found within the transmembrane topology depicting potential antigenic peptides. More so, seven of the predicted epitopes are located outside the transmembrane topology and another one is found on the transmembrane itself; these are non-antigenic in nature.

S/N	Sequence	Start Position	End Position	TMHMM	TMHMM Score
1	GPVFLLLAGIFPFAPPGAAAEPHSLRYNLTVLSWGSVQSGFL TEVHLDGQPFLRCDRQKCRA	4	66	Outside	0.04288
2	RMTLAHI	97	103	Inside	0.66097
3	EGLHSLQEIRVCEI	108	121	Outside	0.25679
4	SSQHFYYDGELFLSQ	129	143	Outside	0.21811
5	KTHYHAMHADCLQELRR	177	193	Inside	0.76815
6	LKSGVVLRR	195	203	Inside	0.56129
7	VPPMVNV	205	211	Outside	0.25411
8	ITVTCRASG	221	229	Inside	0.73449
9	DGVSLSH	242	248	Outside	0.17761
10	YQTWVATRICQ	264	274	Inside	0.87917
11	QRFTCYM	278	284	Inside	0.79020
12	STHPVPSGKVLVLQSHWQTFHVSAVAAAAIFVIIIFYVRCCKK	291	333	Transmembrane	0.00369
13	EGPELVSLQVLDQHPVGT	339	356	Outside	0.07341
14	TQLGFQPLMS	363	372	Outside	0.17418
